# Size-Dependent Electrocatalytic Activity of Gold Nanoparticles on HOPG and Highly Boron-Doped Diamond Surfaces

**DOI:** 10.3390/molecules161210059

**Published:** 2011-12-06

**Authors:** Tine Brülle, Wenbo Ju, Philipp Niedermayr, Andrej Denisenko, Odysseas Paschos, Oliver Schneider, Ulrich Stimming

**Affiliations:** 1 Department of Physics E19, Technische Universität München, James-Franck-Str. 1, 85748 Garching, Germany; 2 Institute of Electron Devices and Circuits, University of Ulm, 89069 Ulm, Germany; 3 Institute of Advanced Study (IAS), Technische Universität München, Lichtenbergstr. 2a, 85748 Garching, Germany; 4 Division 1 of Bavarian Center for Applied Energy Research (ZAE Bayern), Walter-Meißner-Str. 6, 85748 Garching, Germany; 5 nanoTUM, Technische Universität München, 85748 Garching, Germany

**Keywords:** gold nanoparticles, electrocatalytic activity, oxygen reduction reaction, hydrogen evolution reaction, single crystalline diamond

## Abstract

Gold nanoparticles were prepared by electrochemical deposition on highly oriented pyrolytic graphite (HOPG) and boron-doped, epitaxial 100-oriented diamond layers. Using a potentiostatic double pulse technique, the average particle size was varied in the range from 5 nm to 30 nm in the case of HOPG as a support and between <1 nm and 15 nm on diamond surfaces, while keeping the particle density constant. The distribution of particle sizes was very narrow, with standard deviations of around 20% on HOPG and around 30% on diamond. The electrocatalytic activity towards hydrogen evolution and oxygen reduction of these carbon supported gold nanoparticles in dependence of the particle sizes was investigated using cyclic voltammetry. For oxygen reduction the current density normalized to the gold surface (specific current density) increased for decreasing particle size. In contrast, the specific current density of hydrogen evolution showed no dependence on particle size. For both reactions, no effect of the different carbon supports on electrocatalytic activity was observed.

## 1. Introduction

The properties of nanoparticles often show considerable differences as compared to bulk materials. One of the most prominent examples where this change in properties can be observed is gold metal [1,2]. In its bulk form, gold is known as an inert material, with high stability in corrosive environments and low catalytic activity regarding most reactions. However, in the 1980s it was found that supported gold nanoparticles exhibit a surprisingly high catalytic reactivity regarding various reactions such as carbon monoxide and alcohol oxidation in the gas phase [3]. Further investigations showed that only particles with diameters <5 nm displayed high catalytic activities [4,5]. Besides the particle size, the support is also an important parameter which might influence the nanoparticle activity. Nanocrystalline gold supported on oxides has been found to be most reactive in many reactions [2]. In electrocatalysis, size effects were even found on nanostructured surfaces with rather large gold particles: An enhanced electrocatalytic activity for the oxygen reduction reaction (ORR) as compared to extended gold surfaces was reported by several groups [6,7,8,9,10] for particles with diameters >10 nm. It was suggested that the electrocatalytic reaction is very sensitive not only to particle size, but also to structural parameters, such as nanoparticle morphology and preferential surface orientation [11,12]. Substrate material, particle size and deposition method might have a large effect on these parameters. It was further reported that oxide species on the gold surface play an important role in electrocatalytic activity of gold nanoparticles [13,14]. There are very few electrocatalytic investigations on particle sizes <10 nm reported in literature. Chen and Chen [15] observed an increasing activity with decreasing particle size regarding the ORR in alkaline electrolyte for gold particles on glassy carbon in the size range between 0.8 and 1.7 nm in diameter. In contrast to these results, Bron [16] reported no size dependence in acidic electrolyte for particle sizes in the range from 2.7 nm to 42.3 nm supported on carbon black and Guerin *et al.* [17] found for Au nanoparticles on carbon and titania a rapid decrease in ORR activity for particle diameters smaller than 3 nm. Inasaki and Kobayashi [18] reported also decreasing ORR current densities for decreasing particle sizes on carbon black in acidic electrolyte, however the mechanism also changed to the more efficient direct 4-electron pathway for particle diameters <3 nm. In order to further investigate these particle size effects, the controlled variation of particle sizes with narrow size distribution in a size range down to ≈1 nm is essential. Various methods for the preparation of nanoparticles are reported in literature: Methods such as synthesis of nanoparticles using the microemulsion method yield narrow particle size distributions [7,12], but the variation of particle sizes is difficult. Furthermore, remainders of stabilizing molecules might influence the catalytic performance of the synthesized nanoparticles. Sputtering followed by thermal treatment [7,19] is a convenient deposition method allowing adjustment of different coverages, however the distribution of particle sizes is rather broad. Electrodeposition [6,7,11,20,21] is one of the most widely used methods for the deposition of metal particles on boron-doped diamond and other carbon based surfaces. An advantage of this method is the possibility of calculating the amount of deposited metal from the deposition charge. Furthermore, all deposited particles are in electronic contact to the substrate. However, using a single potentiostatic step or cyclic voltammetry, it has proven difficult to prepare nanoparticles of diameter as low as 1–2 nm with a narrow size distribution [7,20]. Enea *et al.* [20] reported a small number of preferential sites on polycrystalline boron-doped diamond on which metal particles are growing fast. This was explained by an inhomogeneous electrical conductivity in the diamond film. Furthermore, diffusional coupling of neighbored particles as well as progressive nucleation might cause a broadening of the size distributions [22]. As was shown by the groups of Penner [23,24] and Plieth [25,26], well-dispersed particles in the mesoscopic range with average particle diameters of 50 nm and above and very narrow particle size distributions on highly oriented pyrolytic graphite (HOPG) and indium tin oxide (ITO) can be obtained using a deposition method consisting of two potentiostatic pulses. With this technique, metal nuclei are formed during a short nucleation pulse with a high overpotential. The nucleation pulse is followed by the growth pulse, where the nuclei slowly grow at low overpotential to their final size. Due to the separation of particle nucleation and growth, it is possible to independently control particle density and particle size. The double pulse technique was recently applied in our group [27] to prepare platinum nanoparticles in the range between 1 nm and 15 nm in height and 5 and 50 nm in apparent radius on monocrystalline epitaxial diamond.

Besides the ORR, also the hydrogen evolution reaction (HER) is an interesting reaction to investigate as earlier results on particle and substrate effects show: Results on gold supported platinum nanoparticles [28,29] showed a large increase in activity regarding this reaction for smaller particle sizes, while the activity of carbon supported platinum nanoparticles was the same as of extended platinum surfaces [27,30]. Furthermore, it was shown that the activity of extended gold surfaces regarding the HER depends on the crystallographic orientation [31,32]. Changes due to different particle sizes could therefore be expected. Even though there are some reports in literature, which show a catalytic activity of gold nanoparticles towards HER [33], this is to our knowledge the first investigation of this reaction on gold nanoparticles with different sizes.

In this work, we used the potentiostatic double pulse deposition method to prepare gold nanoparticles with particle radii down to 1 nm. HOPG and monocrystalline boron-doped diamond were chosen as substrates due to their smooth and homogeneous surface. Furthermore, these carbon based surfaces are reported to interact weakly with metal nanoparticles [7,11,27,30]; therefore we expect the observed effects to be mainly due to particle size. The electrocatalytic activity of the nanostructured surfaces for HER and ORR was investigated using cyclic voltammetry.

## 2. Results

### 2.1. Substrate Characterization

The substrates were characterized electrochemically via cyclic voltammetry and TM-AFM measurements. Both HOPG and diamond show a wide potential window of >2.5 V for HOPG and >3 V for diamond, where the limits at low and high potentials are given probably by hydrogen evolution and oxygen evolution, respectively. The TM-AFM micrographs of diamond show smooth surfaces with roughness rms-values of <0.2 nm, very similar to the equally treated surface in [27]. The freshly cleaved HOPG surface shows the typical atomically smooth surfaces with some steps. The oxidation of the surface leads to a slightly higher roughness rms value of usually <1 nm.

### 2.2. Deposition Transients

**Figure 1:**
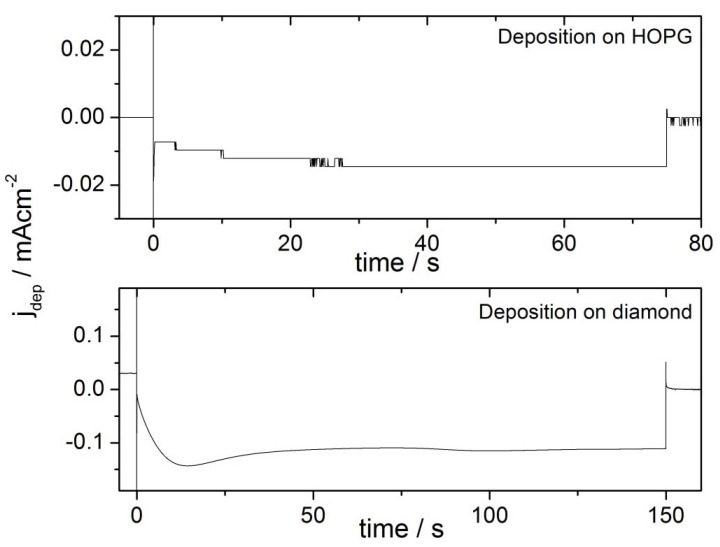
Current transients of double pulse deposition in 0.1 M H_2_SO_4_ + 0.5 mM HAuCl_4_. Note the different current scales. The steps in the deposition transient on HOPG are the result of the limited resolution: Due to high double layer currents associated with the low nucleation potential, a high current range is necessary.

The growth overpotentials for the deposition on the two different substrates were chosen such that the deposition current was constant during the growth pulse. Typical current transients are shown in Figure 1. The nucleation pulse shows on both pictures as vertical line, which denotes the nucleation current and the double layer charging due to the value until it reaches the constant current (−0.014 mA ± 0.002 mA), the deposition current on potential steps. In order to show the growth transients, the current maxima of the higher nucleation currents are cut off in the graph. The growth currents are in both cases constant during the second half of the pulse. In the first half of the pulses, the transients look different. While the deposition current on HOPG increases in absolute diamond goes through a maximum before reaching the final current value (−0.112 mA ± 0.005 mA). Note also the different current scale of the two figures.

### 2.3. AFM Characterization of the Au/HOPG and Au/Diamond Surfaces

**Figure 2:**
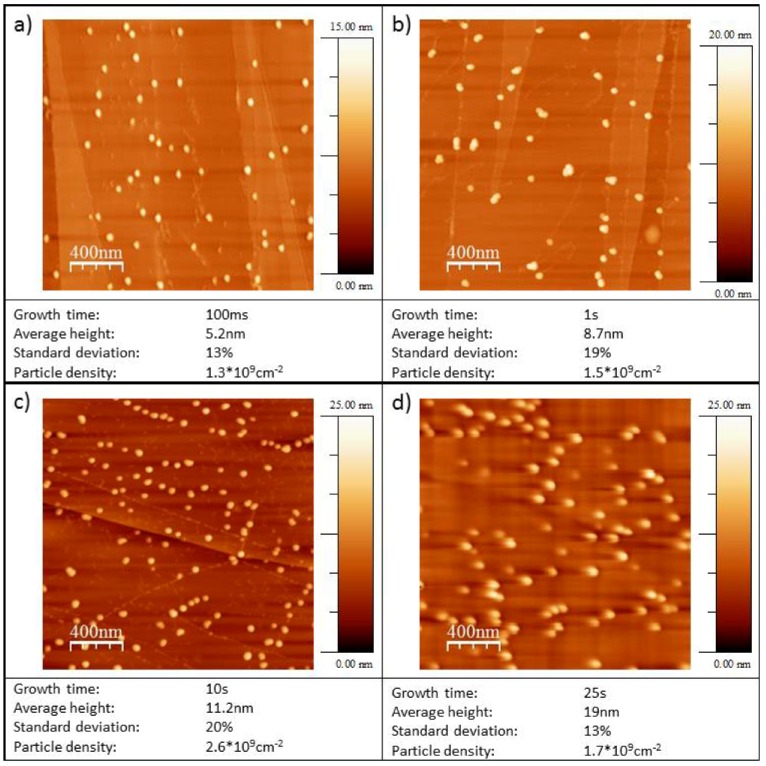
TM-AFM micrographs of Au nanoparticles on HOPG. The deposition parameters were: Nucleation pulse: 31 ms, −40 mV *vs.* NHE, growth pulse: Variable duration as given in micrograph captions, 1.085 V *vs.* NHE.

**Figure 3:**
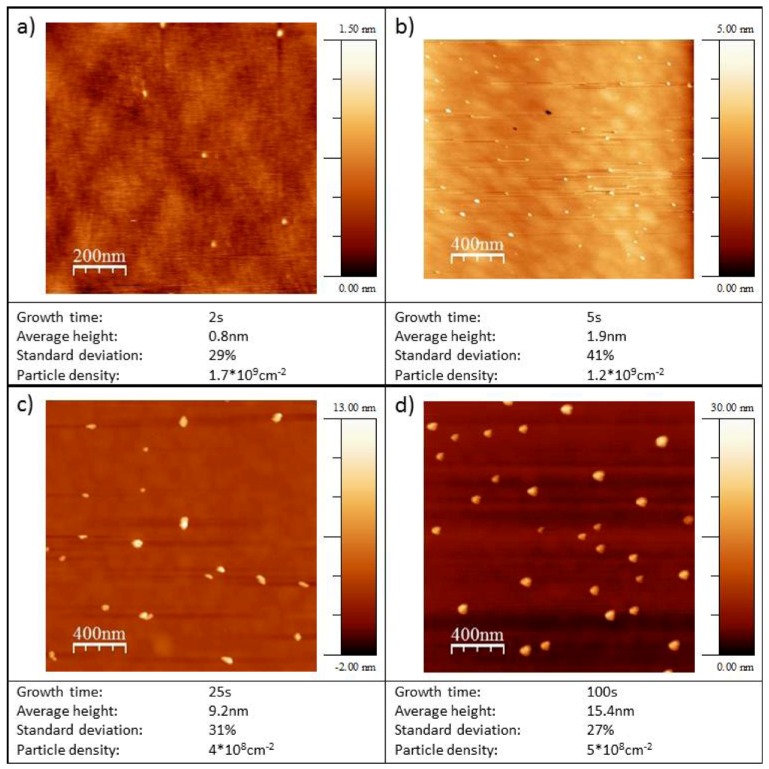
TM-AFM micrographs of Au nanoparticles on diamond. The deposition parameters were: Nucleation pulse: 10 ms, −140 mV *vs.* NHE, growth pulse: Variable duration as given in micrograph captions, 895 mV *vs.* NHE.

Typical micrographs obtained with TM-AFM are shown in Figure 2 for Au/HOPG and Figure 3 for Au/diamond.

**Figure 4:**
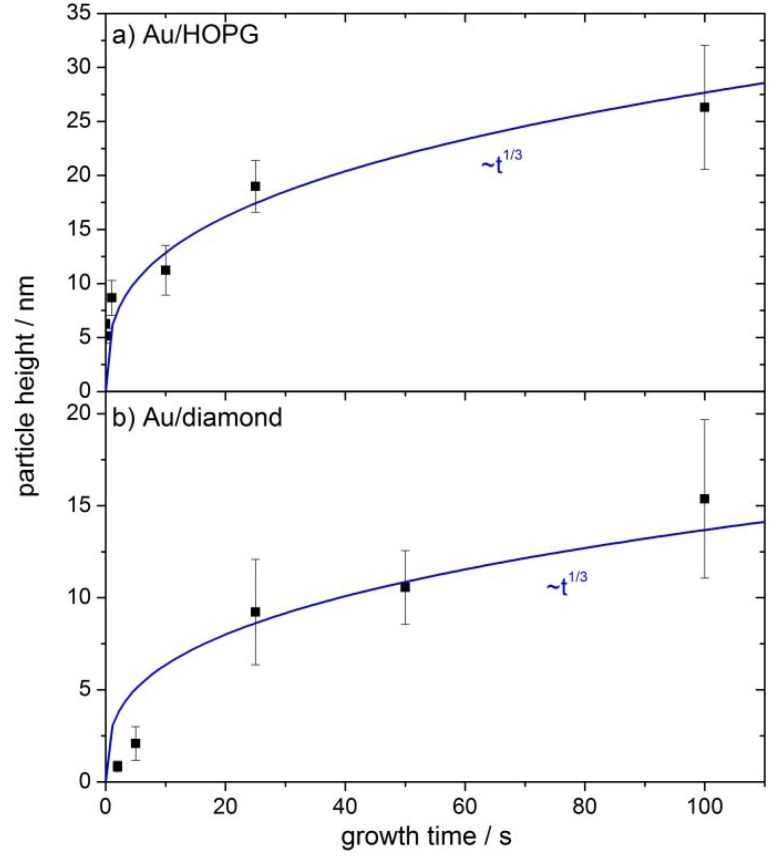
Average particle height versus growth time for Au nanoparticles on HOPG (**a**) and diamond (**b**). The error bars denote the standard deviation σ of particle heights.

The particle densities are about 10^9^ cm^−2^ particles for all growth times t_G_ on both substrates (see Table 1). In all cases the particles are three-dimensional and well-dispersed. In the case of Au/HOPG a preference for particle deposition on step sites is seen on some images (e.g., Figure 2c). For the Au/diamond surfaces, no preferred nucleation sites can be identified. The Au/HOPG surfaces show further some small structures in addition to the bright and round-shaped gold particles. These structures are probably due to the roughening of the surface from the oxidation pulse. Oxidized surfaces without gold particles show similar structures [30]. Since AFM data of supported nanoparticles is much more accurate regarding particle heights than regarding particle radii, only the particle heights are evaluated. The particle radius is usually overestimated when evaluating from AFM-images, due to the convolution of AFM tip and particle. We assume in the following, that the particles are hemispheric, that means the particle radius equals the particle height and is denoted as particle size. However, this assumption does not influence the activity results: The gold area used for the normalization of the catalytic currents is evaluated from the electrochemical evaluation of the gold oxide reduction charge. Average particle heights evaluated in each case from several micrographs are shown in Figure 4a for Au/HOPG and in Figure 4b for Au/diamond. The error bars denote the standard deviation σ of particle heights. The values are given in Table 2. The values for σ are in the range of 13% to 24% for Au/HOPG and between 19% and 44% for Au/diamond. The fitted lines in Figure 4a and Figure 4b show that the particle height increases approximately with t_G_^1/3^.

**Table 1:** Evaluation of TM-AFM measurements of Au/HOPG and Au/diamond-surfaces.

	Growth time t_G_ / s	Particle density / 10^9^ cm^−2^	Average particle height / nm	Standard deviation particle height / %
Au/HOPG	0.01	1.2	6.3	24
	0.1	1.6	5.2	13
	1	1.5	8.7	19
	10	3.1	11.2	21
	25	1.7	19	13
	100	2.3	26.3	22
Au/diamond	2	1.40	0.8	35
	5	0.9	2.1	44
	25	0.4	9.2	31
	50	0.2	10.6	19
	100	0.5	15.4	28

### 2.4. Catalytic Activity Measurements

#### 2.4.1 Oxygen Reduction Reaction

**Figure 5:**
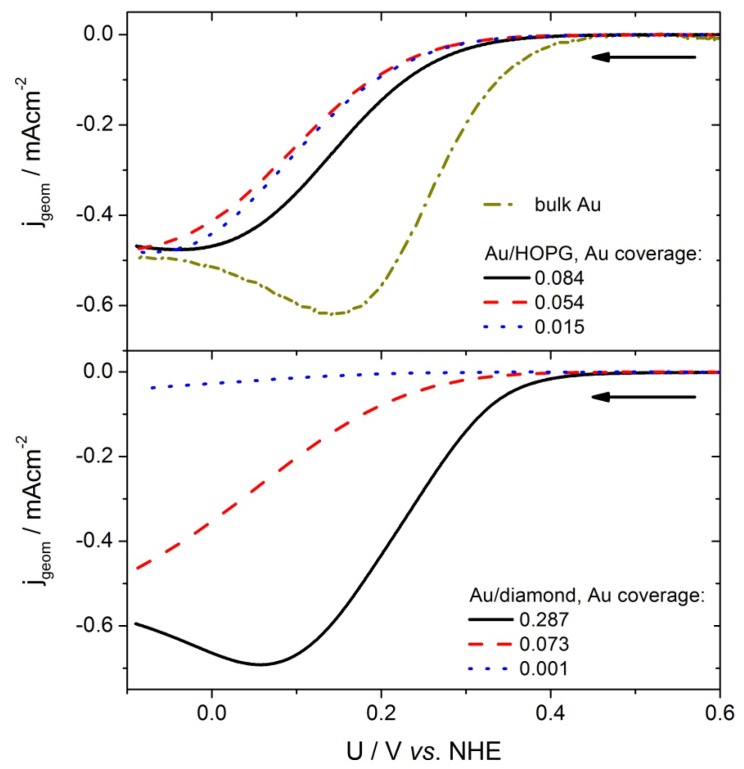
Current-voltage curves of different Au/HOPG and Au/diamond samples as well as the gold bulk sample with decreasing potential in O_2_ saturated 0.1 M H_2_SO_4_. Scan rate: 100 mV/s, the arrows indicate the direction of the potential scan.

The catalytic activity for ORR of the Au/HOPG and Au/diamond surfaces was investigated using current-voltage curves in oxygen saturated 0.1 M H_2_SO_4_. Some typical current-voltage plots for decreasing potential are shown in Figure 5. The currents are normalized to the geometric surface area of the substrate (geometric current density, j_geom_).

**Figure 6:**
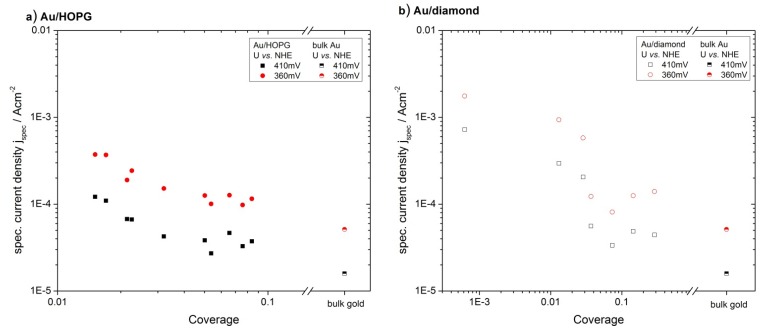
Specific current density for ORR for Au/ HOPG (**a**) and Au/diamond (**b**) evaluated from current-potential curves. The specific current density is increasing with decreasing coverage and decreasing particle height. The data for an extended gold surface was added as a reference.

**Figure 7:**
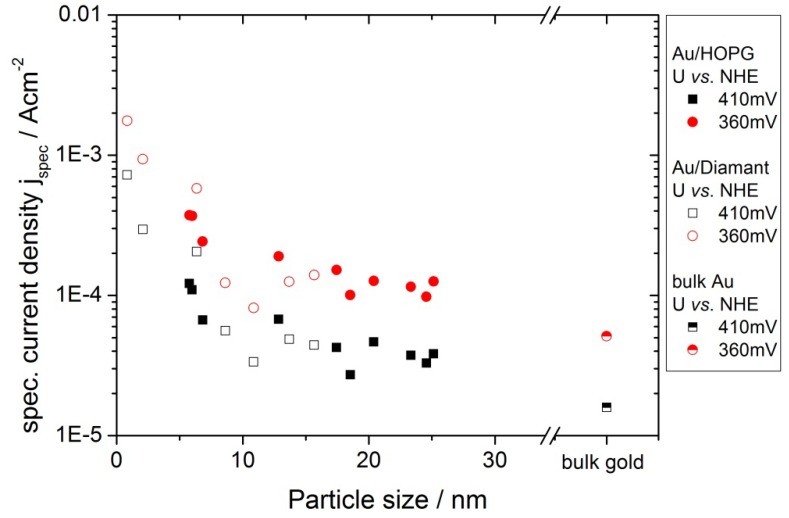
Comparison of the ORR specific current density for Au/HOPG (filled symbols) and Au/diamond (open symbols). The current densities versus particle sizes are comparable for the two substrates. The data for an extended gold surface was added as a reference.

From the onset at ≈0.4V *vs.* NHE, the current increases at first exponentially, as predicted by the Butler-Volmer equation. Then, at higher overpotentials, the limitation of planar diffusion due to an overlap of the depletion zones of the individual nanoparticles becomes the dominating factor, and the current passes through a maximum. The potential at which this maximum occurs, depends on the gold surface area, *i.e.*, the particle size and density. For comparison of the activities for the different amounts of gold, the currents were normalized to the gold surface area obtained from the gold oxide–reduction charge (specific current density, j_spec_). Such normalized current values at two chosen potentials (360 mV, 410 mV) were plotted versus the gold area normalized to the geometrical area of the substrate, which we will denote as coverage from here on. In order to avoid an influence of diffusion limitations, the potentials where chosen such that the geometric current densities are smaller than 1/10 of the diffusion limited current density (see next chapter for further discussion). The results can be seen in Figure 6a for Au/HOPG surfaces and in Figure 6b for Au/diamond surfaces. All plots for the nanostructured surfaces show a higher specific current density as compared to the extended gold surface. Furthermore a clear increase in ORR specific current density for decreasing coverage can be observed. Although the current density values for the Au/diamond substrates scatter more than the values for Au/HOPG, the data clearly shows the same trend. A comparison of the specific current densities on the different substrates is shown in Figure 7. Here, the specific current densities are plotted versus the average particle height, as calculated from the duration of the growth pulse using the fitted lines in Figure 4. For the two shortest deposition times on diamond, the measured values of particle heights were used directly, since the fit is deviating here considerably. For both support materials, the activity increases with decreasing particle size. Furthermore, the particle size-dependent specific current densities on the two surfaces are quantitatively comparable.

#### 2.4.2. Hydrogen Evolution Reaction

**Figure 8:**
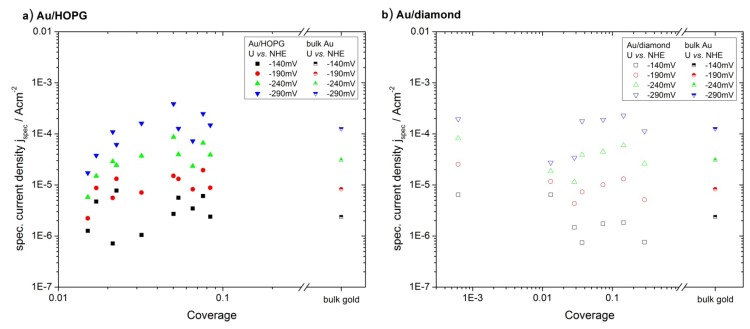
Specific current density for HER for Au/ HOPG (**a**) and Au/diamond (**b**) evaluated from current-potential curves. The specific current density does not show a clear trend for decreasing gold coverage. The data for an extended gold surface was added as a reference.

**Figure 9:**
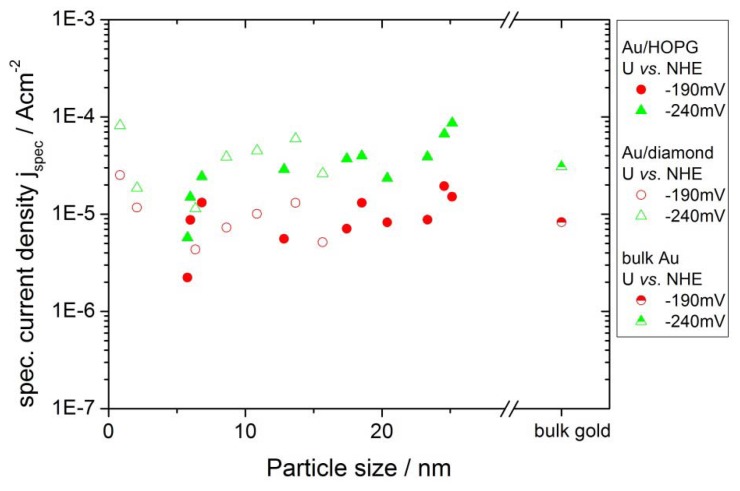
Comparison of the HER specific current density for Au/HOPG (filled symbols) and Au/diamond (open symbols). The current densities versus particle size are comparable for the two substrates. The data for an extended gold surface was added as a reference. For the sake of clarity, only two potentials are displayed.

The catalytic activity for the HER was evaluated in the same way as the ORR measurements. Due to the high concentration and mobility of protons in the electrolyte, the current densities for this reaction usually are not limited by a depletion of reactants. The currents are normalized using the gold area and then plotted for potentials of −140 mV, −190 mV, −240 mV and −290 mV versus gold coverage (Figure 8). For this reaction, the specific current densities versus coverage do not show any clear trend. All nanostructured surfaces as well as the extended gold surface show similar values for the specific current densities. Figure 9 shows that the values for the specific current densities for the two different substrates are quantitatively comparable. The particle sizes in this plot were evaluated the same way as described in the last section. There is further no dependence on the particle size visible.

## 3. Discussion

### 3.1. Nanoparticle Deposition

The double-pulse technique aims at uniform particle growth by separation of the processes of particle nucleation and particle growth. While nucleation of particles is supposed to occur solely during the nucleation pulse, the nuclei grow slowly to their final size during the growth pulse. The results in Table 2 show, that the deposition parameters used in this work result in an approximately constant particle density. The particle density is in the range of (2.1 +/− 1.1) 10^9^ cm^−2^ for Au/HOPG and (0.6 +/− 0.4) 10^9^ cm^−2^ for Au/diamond. Only the lowest growth time on diamond (t_G_ = 2 s) results in a larger particle density by a factor of three, which can be at least partly explained by the difficulty in evaluation of the AFM-images for these small particles (height < 1 nm). The otherwise constant particle density indicates that the nucleation of particles is instantaneous, that means it only takes place during the nucleation pulse. No new nuclei are created during the growth pulse. The particle size increases with the duration of the growth pulse. The current transient of the growth pulse and the relation between particle size and deposition time can give further information about the growth mechanism. Typical deposition transients for the two different substrates are shown in Figure 1. In both cases the current remains constant in the last part of the pulse, which indicates a growth mode as it was described before by the group of Penner [24,34]. In this case, an intermediate case between kinetically controlled growth (see e.g., [27]) and growth controlled by diffusional coupling between neighboring particles applies. The current is in this intermediate case limited by a mix of kinetic and convective transport control [24,34]. The growth of each particle is in this mechanism limited by diffusion, however, the depletion zones of neighboring particles do not overlap. A further indication for this mechanism in this work is the proportionality of particle sizes to t_G_^1/3^ (cf. Figure 4), which directly follows from the constant deposition current and describes a limitation by hemispherical mass transport. From this mechanism, a faster growth of smaller particles can be predicted as well, the size distribution is therefore theoretically narrowing with increasing growth time. The experimental results in this work as well as in literature [24,34] show that the standard deviations are invariant with t_G_. This was ascribed to an additional, yet unidentified mechanism of distribution broadening [24,34]. While the transients of the deposition on the two substrates are both constant in the last part of the pulse, they show some differences in the beginning of the growth pulse. In the case of HOPG as substrate, the current density increases in absolute value, until reaching the final, constant value. For diamond as a substrate, the current density is first going through a maximum, then decreasing to the constant value. The decrease of the current density indicates that there might be some diffusional coupling involved in this part of the pulse. When comparing the scales of the deposition transients on the different substrates, the current density of the deposition on the diamond substrate is much larger. Furthermore, the standard deviations for the particle sizes of the Au/diamond samples are considerably larger than of the Au/HOPG samples (see Table 2). These facts support the assumption that the growth mechanism is in the case of diamond influenced by some diffusional coupling of neighboring particles and indicate an unfavorable choice of deposition parameters. However, smaller growth overpotentials did result in current densities increasing with pulse duration. The difference between the properties of the deposition on the two substrates might result from an influence of the different electrode size on mass transport: While the size of the HOPG electrode was ≈0.13 cm^2^, the size of the diamond electrode was ≈0.008 cm^2^.

For HOPG as a substrate the standard deviations are in the range of 13%–24%. While these values are much smaller than the standard deviations from e.g. single pulse deposited particles [22,30], we did not achieve values of <10% as reported by Liu *et al.* [24,34] for particles in the range from 50 nm to 2 μm prepared using the double pulse method. However, we succeeded to apply the double pulse method to control particle size and particle density for much smaller nanoparticles in the range down to 4 nm on HOPG and to <1 nm on diamond, with a comparably narrow size distribution.

While Au/diamond surfaces with particle sizes down to <1 nm were successfully prepared, we did not achieve Au nanoparticles of comparable small sizes on HOPG. A further reduction of growth time did not result in smaller particles in the case of HOPG as substrate, even for t_G_ = 0, *i.e.*, when applying solely the nucleation pulse and no growth pulse, no particles smaller than 4 nm were achieved. We therefore think that the compared to the particles on diamond large particle sizes are due to the long nucleation pulse.

### 3.2. Activity of Supported Au Nanoparticles for Oxygen Reduction

Figure 6 and Figure 7 show a clear dependence of the oxygen reduction current on the particle size. An effect of the support material cannot be observed, the Au surface specific current densities are quantitatively comparable for the Au/HOPG and Au/diamond surfaces. All investigated Au/carbon nanostructured surfaces show an enhanced specific current density as compared to an extended gold surface. This includes gold particles with a radius of up to 26 nm. Similar results can be found in literature for electrocatalytic measurements on gold nanoparticles: An enhanced activity towards ORR for rather large (11 nm–60 nm) nanoparticles supported on carbon based substrate materials such as boron doped diamond and carbon nanotubes or on gold surfaces as compared to the bulk material has been reported in both alkaline [6] and acidic [7,8,9,10] electrolyte. These results suggests a size dependence already at particle sizes in the range of r > 10 nm, where particles are expected to act like bulk material: The size range of nanoparticles that exhibit properties due to the quantum size effect is predicted to be <10 nm, severe changes in properties due to these effects were usually observed for particle sizes <3 nm [1]. For particle sizes >10 nm it is therefore improbable that the higher activity is due to quantum size effects. An alternative explanation might be the influence of surface structures such as the number of low coordinated sites or the orientation of surface facets. El-Deab *et al. * [11] and Hernandez *et al.* [12] explained differences in activity and reaction pathway in alkaline solution with differences in the orientation of the surface, which is strongly influenced by the substrate. Hernandez further showed that nanoparticles with a high ratio of surfaces with (100) facets have the highest catalytic activity. This is in accordance with the catalytic activity on extended single crystal surfaces in alkaline electrolyte: While (100) facets are most active, Au(111) is the least active plane [35]. Similar results were found on gold single crystal surfaces in acidic electrolyte: Au(100) showed a higher activity towards ORR than Au(110) or Au(111) [36].

For decreasing particle size, we observed an increase in surface specific activity in acidic solution; the activity for the smallest particle sizes is more than an order of magnitude higher than the activity of the extended gold surface and large gold particles. The same trend was found by Chen and Chen [15] for the ORR on Au nanoparticles supported on glassy carbon in alkaline electrolyte, where an increasing activity for decreasing particle diameter in the range of 0.8 nm to 1.7 nm was found. Other results are in contrast to this: Bron [16] found for particle sizes in the range between 2.7 nm and 42 nm on carbon black no size effect in acidic electrolyte, for gold nanoparticles on carbon, the activity decreases for diameters <3 nm with decreasing particle size [17]. We think that the differences in the results might be due to the strong dependence of the reaction on structural properties of the nanoparticles: in all experiments different deposition methods and different supports where used. Remains of chemical particle synthesis might in some cases influence the measured particle activity or the form of binding with the substrates.

An effect that has to be considered is a possible current limitation by mass transport. The current voltage curves were measured without using a method to enhance the diffusion of reactants such as rotating disc or flow cell measurements. Therefore, for the extended gold surface, a planar depletion layer forms quickly and might reduce the measured current considerably at high current densities, while the effect on Au/HOPG and Au/diamond nanostructured surfaces is significantly smaller. However, the geometric current densities as shown in Figure 5 at low overpotentials (360 mV, 410 mV *vs.* NHE) are below 10% of the expected diffusion limited current density of ≈0.5 mAcm^−2^. This was estimated using the expression:

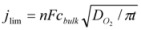

where n = 2 the number of transferred electrons for the two-electron process, F the Faraday constant, c_bulk_ ≈ 1 × 10^−6^ mol cm^−3^ the bulk concentration of oxygen in solution and D_O2_ ≈ 2 × 10^−5^cm^2^s^−1^ [37,38] and is in good agreement with the measured limits observed in Figure 5 at large overpotentials. Therefore we don’t think the decrease in measured current density for increasing coverage is due to transport limitations.

Another important point for the interpretation of our results is the fact that we cannot make any statement on the reaction pathway of the oxygen reduction. Next to high current densities, it is preferable to achieve an efficient conversion of oxygen via the 4-electron, direct reaction pathway, instead of the indirect, 2-electron pathway which usually predominates on gold surfaces in acidic solutions [36,39]. However, we focused in this work on the control of particle size and density in order to investigate if size effects can be observed. A detailed investigation of the reaction pathway on these nanoparticles is subject of future work. 

### 3.3. Activity of Supported Au Nanoparticles for Hydrogen Evolution

In contrast to the results on the ORR, the Au nanoparticles did not show any size effect on the activity for HER in the range of accuracy of the measurements reported here. This is a surprising observation for particle sizes down to r < 1 nm due to the considerable electronic changes in the structure of very small nanoparticles, as compared with larger particles or the bulk material. An effect on the catalytic activity could therefore be expected [1]. Furthermore, the electrolytic hydrogen evolution was reported to be a very structure-sensitive process [32]. On different vicinal surfaces of gold single crystals, the exchange current density was found to differ up to almost an order of magnitude [31]. However, the reaction appears to be much less sensitive to changes in particle size than the ORR. The reason for this can probably be found in the interaction of reactants, products and intermediates with the nanoparticle surface. As for the ORR, the specific activities of the gold nanoparticles are similar for the two different supports. The substrate is therefore not influencing the activity of the nanoparticles towards HER.

## 4. Experimental Section

### 4.1. General

Metal deposition, reactivity measurements and electrochemical characterization were performed in glass cells with standard three electrode arrangement using a potentiostat-galvanostat (Autolab PGSTAT 30). The solutions were prepared from H_2_SO_4_ (96% Merck, suprapur), HAuCl_4_ hydrate (99.999%, Aldrich) and CuSO_4_ (99.999% Alfa Aesar) with deionized water obtained from a Millipore-Milli-Q (18.2 MΩcm, 3 ppm total organic carbon). Aqua regia was prepared using HNO_3_ (65% Merck, p.a.) and HCl (32% Merck, extra pure) at a volume ratio of 1:3. Peroxymonosulfuric acid (Caro’s acid) was prepared with H_2_SO_4_ (95–97% Merck, p.a.) and H_2_O_2_ (33% Merck, p.a.) at a volume ratio of 1:1. All glassware was cleaned in Caro’s acid and rinsed extensively with deionized water. Hg/Hg_2_SO_4_ reference electrodes (Schott, B3610) in 0.1M H_2_SO_4_ were used in all experiments. Gold wires served as counter electrodes. HOPG was purchased from Mikromasch, USA (grade ZYB). A planar Au substrate (11 × 11 mm^2^, Schroer GmbH) was used as a reference surface. The substrate was not subjected to annealing, in order to keep the polycrystalline orientation of the surface. Surface images were obtained using a Veeco Multimode Electrochemical Scanning Tunneling Microscope/Atomic Force Microscope (EC-STM/AFM) system using atomic force microscopy in tapping mode (TM-AFM) under dry conditions. Phosphorus (n) doped Si cantilevers (RTESPA, Bruker) were used. The AFM images were evaluated with WSxM 5.0 image analysis software (Nanotec Electronica S.L. [40]) to quantify the resulting surface morphology and gold particle coverage and height.

### 4.2. Preparation of the HOPG and Diamond Electrodes

The HOPG surface was prepared prior to each experiment by cleaving the surface with an adhesive tape. For the electrochemical measurements the HOPG substrate was partly covered with Teflon tape in order to provide a well-defined surface-area of 0.13 cm^2^ that is in contact with the electrolyte. To enhance the number of edge and defect sites and therefore increase the adhesion of metal particles to the surface, the graphite surface was electrochemically oxidized by applying a 100 μs long potential pulse with 5.7 V *vs.* NHE in 0.1 M H_2_SO_4_.

Diamond layers were grown by chemical vapor deposition (CVD) in a microwave plasma reactor on highly polished (100)-oriented surfaces of a high-pressure high-temperature synthetic type 1b diamond crystal by Sumitomo. The CVD grown layer at the surface was boron-doped using a solid doping source and approx. 20 nm thick. The concentration of the boron acceptors was approx. 4 × 10^20^ cm^−3^. Details on diamond growth, doping evaluation and initial characterization can be found elsewhere [41,42,43]. An oxygen termination of the surface was achieved by wet chemical oxidation in hot Caro’s acid for 10 min [42]. Before each experiment, the diamond surface was cleaned and prepared as described in a previous publication [27]. A surface area of 0.008 cm^2^ was in contact with the electrolyte.

### 4.3. Electrochemical Gold Deposition and Characterization of the Nanostructured Surfaces

Deposition of gold was performed from an aqueous solution of 0.5 mM HAuCl_4_ in 0.1 M H_2_SO_4_. The solution was deaerated using argon gas and unstirred during the deposition. The particles were deposited using the potentiostatic double pulse technique. The deposition parameters are summarized in Table 2. Before and after Au deposition, the potential was kept at 1.16 V *vs.* NHE to avoid further deposition.

**Table 2:** Parameters for double pulse deposition of gold on HOPG and diamond.

	Nucleation pulse		Growth pulse	
	Potential *vs.* NHE	Pulse duration	Potential *vs.* NHE	Pulse duration
HOPG	−0.04 V	31 ms	1.085 V	1 s–100 s
Diamond	−0.14 V	10 ms	0.895 V	2 s–150 s

The deposition parameters were chosen for both substrates such that the particle density was ≈10^9^ cm^−2^ and the current during the growth pulse was approximately constant in order to achieve a narrow distribution of particle sizes. The data in Table 2 shows, that the deposition overpotentials for the deposition on diamond are chosen to be higher than for the deposition on the HOPG surface. This is necessary due to the semiconducting properties of the diamond sample: due to the potential drop across the depletion zone at the surface of the substrate, the actual overpotential at the electrode/electrolyte interface is much smaller [27]. After deposition the samples were removed from the cell and rinsed with deionized water to avoid spontaneous Au deposition.

The Au/HOPG and Au/diamond samples were characterized in Ar purged 0.1 M H_2_SO_4_ with no stirring during the measurements. The active surface area of the gold nanoparticles was estimated by integrating the current of the reduction of the gold surface oxide monolayer from cyclic voltammetry between −0.29 V *vs.* NHE and 1.66 V *vs.* NHE with a scan rate of 100 mV/s. This method is based on the results of Michri *et al.* [44], who state that a complete monolayer (400 μCcm^−2^) of reversibly adsorbed oxygen atoms is existent at the potential corresponding to the minimum in the current in the gold oxidation region. This result was affirmed by Kondo *et al.* [45] using in situ surface X-ray scattering techniques. For the Au/HOPG samples, the active surface area was additionally determined using the charge of the underpotential deposition (upd) of copper. This was done using cyclic voltammetry in 10 mM CuSO_4_ + 0.1 M H_2_SO_4_ with a scan rate of 10 mV/s. A charge of 400 μCcm^−2^ for a complete monolayer was assumed in the calculations. The results were comparable to the results obtained from the reduction of the gold surface oxide.

The activity measurements with respect to the HER were performed in hydrogen saturated 0.1 M H_2_SO_4_. The current of hydrogen evolution was obtained from current-voltage curves with a scan rate of 5 mV/s. The activity measurements with respect to the ORR were done in oxygen saturated 0.1 M H_2_SO_4_ using current voltage curves with a scan rate of 100 mV/s. The large scan rate was chosen in order to prevent a too early onset of the depletion of oxygen molecules at the electrode surface.

## 5. Conclusions

Gold nanoparticles with a narrow size distribution for application in electrocatalytic measurements were prepared on HOPG and single crystalline, boron doped diamond surfaces. The particles were deposited electrochemically using the potentiostatic double pulse technique. Average particle radii were in the range of 5 nm to 30 nm on HOPG and between <1 nm and 15 nm on boron doped diamond surfaces. The Au/HOPG and Au/diamond surfaces were examined for their electrocatalytic activity regarding oxygen reduction and hydrogen evolution in 0.1 M H_2_SO_4_ saturated with oxygen and hydrogen, respectively. An influence of the substrate could not be observed: For both reactions the measured current densities for the same particle sizes were quantitatively comparable for the two substrates. For the activity towards ORR a size effect was observed: (i) for all particle sizes, the specific current densities were larger for the nanostructured Au/HOPG and Au/diamond surfaces than for the extended gold surface; (ii) The activity was increasing with decreasing particle sizes. The activity for the smallest particles with a radius of 1–2 nm was about an order of magnitude higher than the activity of an extended gold surface and large particles. For the activity of the Au/HOPG and Au/diamond samples towards HER, no effect of particle size was found.
